# The diagnostic value of advanced tracer kinetic models in evaluating high grade gliomas recurrence and treatment response using dynamic contrast-enhanced MRI

**DOI:** 10.3389/fonc.2025.1536122

**Published:** 2025-04-16

**Authors:** Jianan Zhou, Zujun Hou, Xiuqi Guan, Zhengyang Zhu, Han Wang, Cong Wang, Wei Luo, Chuanshuai Tian, Huiquan Yang, Meiping Ye, Sixuan Chen, Xin Zhang, Bing Zhang

**Affiliations:** ^1^ Department of Radiology, Nanjing Drum Tower Hospital Clinical College of Nanjing Medical University, Nanjing, China; ^2^ Institute of Medical Imaging and Artificial Intelligence, Nanjing University, Nanjing, China; ^3^ Medical Imaging Center, Department of Radiology, Nanjing Drum Tower Hospital, Affiliated Hospital of Medical School, Nanjing University, Nanjing, China; ^4^ The Second Affiliated Hospital and Yuying Children's Hospital, Wenzhou Medical University, Wenzhou, China; ^5^ FISCA Healthcare Co., Ltd., Nanjing, China; ^6^ Nanjing Center for Applied Mathematics, Nanjing, China; ^7^ School of Electronics and Information Engineering, Suzhou Vocational University, Suzhou, China

**Keywords:** high grade glioma, dynamic contrast-enhanced, tracer kinetic model, treatment response, recurrence

## Abstract

**Background:**

The purpose of this study was to investigate the diagnostic value of advanced tracer kinetic models (TKMs) in differentiating HGGs recurrence and treatment response.

**Methods:**

A total of 52 HGGs were included. DCE images were analyzed using the following TKMs: distributed parameter (DP), tissue homogeneity (TH), Brix’s two-compartment (Brix) and extended-Tofts model (ETM), yielding the following parameters: cerebral blood flow (CBF), mean transit time (MTT), plasma volume (V_p_), extravascular volume (V_e_), vascular permeability (PS) and first-pass extraction ratio (E) in advanced TKMs (DP, TH and Brix); K^trans^, V_e_, V_p_ and K_ep_ in ETM. Two delineation methods were conducted (routine scans and parameter heat maps). The differences between two MRI scanners were compared. Mann–Whitney U test was used to assess the difference of parameter values. Diagnostic performance was assessed using the method of the receiver operating characteristic (ROC) curves, with the areas under the ROC curves (AUC) to determine the discriminating power of DCE parameters between recurrent tumor group and treatment response group . *P*<0.05 indicates statistical significance.

**Results:**

The difference on the normalized kinetic parameter value (with respect to contralateral normal-appearing white matter) between two MRI scanners was statistically insignificant (*P*>0.05). MTT and V_p_ of advanced TKMs were higher in recurrent than in treatment response group (*P*<0.05). For ROI delineated on parameter heat maps, MTT(DP) attained the best performance with AUC 0.88, followed by MTT(TH) and V_p_ (DP, Brix) with AUCs around 0.80 (0.81, 0.80, 0.79 respectively). The best performance in ETM was V_p_ (AUC = 0.73).

**Conclusion:**

MTT (DP, TH), and V_p_ (DP, Brix) could be potential quantitative imaging biomarkers in distinguishing recurrence and treatment response in HGGs.

## 1 Introduction

High-grade gliomas (HGGs) are the most common primary brain malignancies, and the first line of care consists of surgical resection, radiation therapy (RT), and chemotherapy (CTX) (1). The extent of resection has been validated as a prognostic marker (2). After maximal safe resection, the standard therapy (Stupp protocol) remains RT with concurrent temozolomide (TMZ) 75 mg/m2/day for 6 weeks and maintenance TMZ (150–200 mg/m2/day × 5 days for 6 cycles) (3). In spite of the survival benefit associated with adjuvant radiation and chemotherapy, the majority of HGGs patients relapse after initial therapy.

Contrast-enhanced MRI is the gold standard imaging method in detecting HGGs and defining their extension, and is recommended as the standard method for evaluating treatment response in the Response Assessment in Neuro-Oncology (RANO) 2.0 criteria (4, 5). However, using conventional MRI alone, sensitivity and specificity could be limited in distinguishing tumor recurrence from radiation-induced brain injury (RIBI), including pseudoprogression (PsP) and radiation necrosis (RN), collectively known as treatment response, which may both present with enlarging contrast-enhancing lesions or expanding edema. Although advanced imaging techniques have been investigated to improve diagnostic accuracy, the temporal overlap of imaging features between PsP and recurrence (both predominantly occurring 3–6 months post-treatment) complicates definitive diagnosis based on single-timepoint imaging assessments, which relies on multiple follow-ups imaging evaluations, thereby prolonging the diagnostic timeline (6). The incidence of RN could be up to 24% (7), and the incidence of PsP could be up to 32.3% in HGGs patients treated with standard regimen (8–13), which is related to the radiation dose and the volume of brain tissue irradiated (14). The distinction between recurrent tumor and treatment response has important implications for further treatment.

Advanced MRI techniques have been developed to aid in differentiating PsP from true recurrence (15) and a promising representative is dynamic contrast-enhanced (DCE) MRI, which quantitatively measures tissue microcirculation through analyzing the time-intensity curve using tracer kinetic models (TKMs). A variety of TKMs, such as conventional TKMs (e.g., Tofts model and extended-Tofts model [ETM]) and advanced TKMs (e.g., Brix’s conventional two-compartment model [Brix], tissue homogeneity model [TH] and distributed parameter [DP] model), have been proposed and investigated in evaluating glioma diagnosis and treatment response, as detailed in a recent review paper (16). A key difference between conventional and advanced TKMs lies in the characterization of tracer molecular transport type in tissue microenvironment. Two types of transport are accounted for in advanced TKMs, namely the transport due to blood flow within the intravascular space and the exchange through vessel wall between the intravascular space and the extravascular space, which is separately modelled as blood flow and vessel permeability. In contrast, only one type of transport is modelled in conventional TKMs. Tofts model is the only single-compartment model, which assumes that the volume of extravascular extracellular space (EES) is much larger than that of intravascular plasma space (IVPS), hence the compartment of IVPS is neglected in the Tofts model. In the above review paper, inconsistent findings in different studies were highlighted and appraised, and advantages of advanced TKMs over conventional TKMs were discussed, but need to be validated in more studies.

In this study, we attempted to investigate the diagnostic value of advanced TKMs and identify potential quantitative imaging biomarkers in differentiating HGG recurrence from treatment response.

## 2 Materials and methods

### 2.1 Participants enrollment

**Figure 1:**
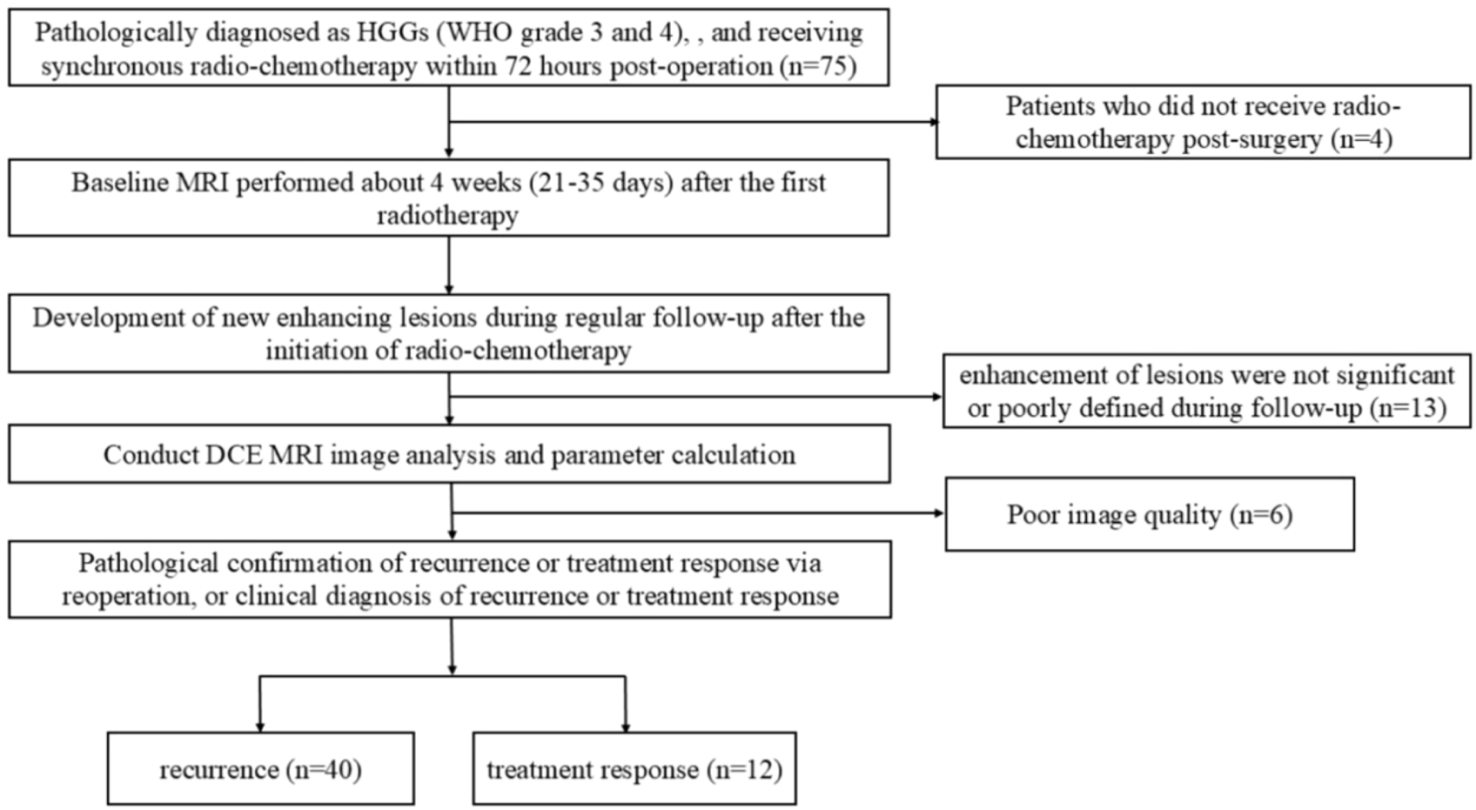
Flowchart of patient inclusion.

This retrospective study was approved by the institutional review board and performed in accordance with the Declaration of Helsinki. Patients in this study were enrolled between December 2022 to May 2024. The requirement for informed consent was waived. The inclusion criteria were as follows: (1) Pathologically diagnosed as HGGs (WHO grade 3 and 4) according to the 2021 World Health Organization (WHO) criteria, with no prior tumor-related treatment before surgery, and receiving synchronous radio-chemotherapy within 72 hours post-operation; (2) Baseline MRI performed about 4 weeks (21-35 days) after the first radiotherapy; (3) Follow-up scans including T_1_WI, T_2_WI, T_2_-FLAIR, T_1_CE (T_1_ contrast-enhanced), and DCE; (4) Development of new enhancing lesions during regular follow-up after the initiation of radio-chemotherapy; (5) Pathological confirmation of recurrence or treatment response via reoperation, or clinical diagnosis of recurrence or treatment response during regular follow-up according to the RANO 2.0 criteria (an increase of ≥25% in the product of the two perpendicular diameters of the maximum cross-section of enhancing lesions compared to baseline, or the emergence of new enhancing lesions outside the radiation target area indicating progression). The exclusion criteria included: (1) Patients who did not receive radio-chemotherapy post-surgery; (2) Cases where enhancement of lesions was not significant or poorly defined during follow-up; (3) Poor image quality due to significant patient motion, resulting in failed DCE data processing. The process for patient inclusion is illustrated in **Figure 1**.

### 2.2 MRI examinations

The data acquisition machines used in this study included the Philips Ingenia CX 3.0 T MRI scanner and the United Imaging uMR790 3.0 T MRI scanner (equipped with a 32-channel head coil). The contrast agent used was Gadobutrol injection (Oniyin, GE Healthcare), administered at an injection rate of 3.5 mL/s and a dosage of 0.2 mL/kg. The scanning sequences included T_1_WI, T_2_WI, T_2_-FLAIR, T_1_CE, and DCE. The DCE sequence included three precontrast sequences with: TR/TE (3.47 ms /1.9 ms), FOV (240 ×220 mm2), slice thickness (5 mm), number of phases (5), flip angles (5°,10°,15°), and the postcontrast dynamic sequence with the same scanning parameters except for number of phases (90) and flip angle (13°). The temporal resolution was 4 seconds, with total duration 6 mins. Specific scanning parameters were shown in **Supplementary Table SA1**.

### 2.3 Image analysis

**Figure 2:**
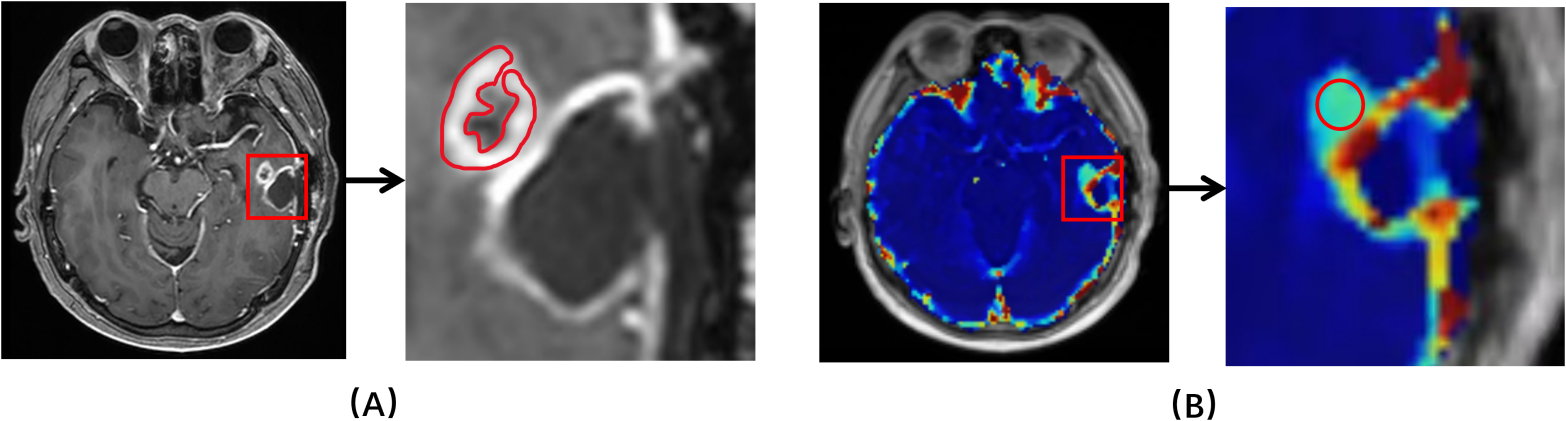
An example of ROIs drawn. **(A)** Based on structural T1 contrast-enhanced MRI. **(B)** Based on kinetic parameter maps.

DCE images were analyzed by a commercially available software for DCE data analytics (MItalytics, FISCA Healthcare, Singapore), using the following TKMs: DP, TH, Brix and ETM, yielding the following parameters: cerebral blood flow (CBF), mean transit time (MTT), plasma volume (V_p_), extravascular volume (V_e_), vascular permeability (PS) and first-pass extraction ratio (E) in advanced TKMs (DP, TH and Brix); K^trans^, V_e_, V_p_ and K_ep_ in ETM. All of the DCE analysis models were available with the software. Images were registered when evident movement was observed among the dynamic scans. The software used the method of variable flip angle to compute tissue ${\text{T}}_{1}$ value and estimated tracer concentration by the difference in longitudinal relaxation rates between postcontrast and precontrast ( $r_{1}C=\frac{1}{{\text{T}}_{1}^{c}}-\frac{1}{{\text{T}}_{1}^{0}}$ , where $r_{1}$ denotes the longitudinal relaxivity and assumes 4.0 ${\text{s}}^{-1}{\text{mM}}^{-1}$ for the contrast agent used in this study (17, 18). Regions of interest (ROI) delineation was performed independently by two neuroradiologists (ZZ and JZ with 9 and 7 years of experience in neuro-radiography). Two types of delineation methods were conducted, one with reference to the routine clinical scans (based on enhanced lesion and areas of necrotic, cystic and hemorrhages were avoided) and the other with account of parameter heat maps (based on the region of highest signal, no less than 15 voxels). **Figure 2** showed an example with two types of ROIs drawn. The observers were blinded to pathohistological results. After manual delineation of all datasets, every case was read by both observers to ensure high-quality measurements. Different opinions were resolved by consensus, with a third observer when necessary. ROIs for contralateral normal-appearing white matter were also delineated. Due to limitation in spatial resolution, substantial partial volume corruption could be arisen in the carotid. Hence, a surrogate for the artery input function (AIF), namely the concentration time course in the sagittal sinus, was utilized in this study.

The DCE software provided comprehensive tools for DCE data analytics, including semi-quantitative analysis, conventional TKMs and advanced TKMs. In this study, the following four TKMs were employed for comparison. For completeness, the equations of four TKMs were described as follows. Let $C_{tiss}\left(t\right)$ and $C_{a}\left(t\right)$ denote the concentration of contrast agent in the tissue of interest and in the arterial plasma respectively. By assuming that the capillary-tissue system is stationary and linear, these two variables can be related as follows:


$$C_{tiss}\left(t\right)=R\left(t\right)⊗C_{a}\left(t\right)$$

where R(t) stands for the impulse residue function and ⊗ the convolution operator. The residue functions of four TKMs were listed below.

ETM:


$${\text{R}}_{\text{ETM}}\left(t\right)={\text{v}}_{\text{p}}\delta \left(t\right)+{\text{K}}^{\text{trans}}\text{exp}\left(-\frac{{\text{K}}^{\text{trans}}}{{\text{v}}_{\text{e}}}t\right)$$

Brix (Equations 1a–1c):


(1a) $${\text{R}}_{\text{Brix}}\left(t\right)={\text{ F}}_{\text{p}}\left[A\;\text{exp}\left(\text{α }t\right)+\left(1-A\right)\text{exp}\left(\text{β }t\right)\right]$$

(1b) $$\left(\begin{array}{c} \text{α}\\ \text{β} \end{array}\right)=\frac{1}{2}\left[-\left(\frac{\text{PS}}{{\text{v}}_{\text{p}}}+\frac{\text{PS}}{{\text{v}}_{\text{e}}}+\frac{{\text{F}}_{\text{p}}}{{\text{v}}_{\text{p}}}\right)\pm \sqrt{{\left(\frac{\text{PS}}{{\text{v}}_{\text{p}}}+\frac{\text{PS}}{{\text{v}}_{\text{e}}}+\frac{{\text{F}}_{\text{p}}}{{\text{v}}_{\text{p}}}\right)}^{2}-4\frac{\text{PS}}{{\text{v}}_{\text{e}}}\frac{{\text{F}}_{\text{p}}}{{\text{v}}_{\text{p}}}}\right]$$

(1c) $$\text{A}=\frac{\text{α}+\frac{\text{PS}}{{\text{v}}_{\text{p}}}+\frac{\text{PS}}{{\text{v}}_{\text{e}}}}{\text{α}-\text{β}}\;$$

where ${\text{F}}_{\text{p}}$ stands for the flow rate of blood plasma through the IVPS and is generally denoted as CBF when applied to cerebral perfusion imaging.

DP model (Equation 2):


(2) $${\text{R}}_{\text{DP}}(t)={\text{F}}_{\text{p}}\left\{\begin{array}{l} \text{u}\left(t\right)-\text{u}\left(t-\frac{{\text{v}}_{\text{p}}}{{\text{F}}_{\text{p}}}\right)+\\ \text{u}\left(t-\frac{{\text{v}}_{\text{p}}}{{\text{F}}_{\text{p}}}\right)\left\{1-\text{exp}\left(-\frac{\text{PS}}{{\text{F}}_{\text{p}}}\right)\left[1+\int_{0}^{t-\frac{{\text{v}}_{\text{p}}}{{\text{F}}_{\text{p}}}}\text{exp}\left(-\frac{\text{PS}}{{\text{v}}_{\text{e}}}\text{τ}\right)\sqrt{\frac{\text{PS}}{{\text{v}}_{\text{e}}}\;\frac{\text{PS}}{{\text{F}}_{\text{p}}\;}\frac{1}{\text{τ}}}{\text{ I}}_{1}\left(2\sqrt{\frac{\text{PS}}{{\text{v}}_{\text{e}}}\;\frac{\text{PS}}{{\text{F}}_{\text{p}}}\text{τ }}\right)\text{dτ}\right]\right\} \end{array}\right\}$$

where *u*(*t*) denotes the Heaviside unit-step function and *I*_1_ is the modified Bessel function.

TH model (Equation 3):


(3) $${\text{R}}_{\text{TH}}(t)\;=\;{\text{F}}_{\text{p}}\{\begin{array}{c} \left[1-\text{exp}\left(-\frac{\text{PS}}{{\text{F}}_{\text{p}}}\right)\right]\text{exp}\left\{-\frac{{\text{F}}_{\text{p}}}{{\text{v}}_{\text{e}}}\left[1-\text{exp}\left(-\frac{\text{PS}}{{\text{F}}_{\text{p}}}\right)\right]\left(\text{t}-\frac{{\text{F}}_{\text{p}}}{{\text{v}}_{\text{p}}}\right)\right\} \end{array}\}\;$$

After the analysis of a TKM, a few other parameters can be derived as follows. The tracer mean transit time (MTT) can be given by the central volume principle (Equation 4)


(4) $$\text{MTT}=\frac{{\text{v}}_{\text{p}}+{\text{v}}_{\text{e}}}{{\text{F}}_{\text{p}}}$$

The first-pass extraction fraction (E) from the IVPS to EES can be evaluated as Equation 5


(5) $$\text{E}=1-\text{exp}\left(-\frac{\text{PS}}{{\text{F}}_{\text{p}}}\right)$$

The primary difference between ETM and another three advanced TKMs lies in that the latter utilizes parameter ${\text{F}}_{\text{p}}$ to account for tracer intravascular transport and parameter PS for tracer exchange between IVPS and EES, whereas the former describes tracer transport using one parameter ${\text{K}}^{\text{trans}}$ , which is in principle a mixture between ${\text{F}}_{\text{p}}$ and PS. As for the difference among the advanced TKMs, it largely pertains to how the tracer distributes within a compartment, which is generally described as compartmental (meaning that tracer is well-mixed in the compartment) or distributed (indicating that the tracer distribution is a function of both time and space). Brix assumes both IVPS and EES to be compartmental; DP assumes both IVPS and EES to be distributed; and TH assumes EES to be compartmental and IVPS to be distributed. Interested readers can refer to the review paper (19) for more details of the different tracer kinetic models.

### 2.4 Statistical analysis

The data was partitioned into tumor recurrent group and treatment response group, based on histopathologic and follow-up imaging and clinical results. For each patient, the parameter values of all voxels within the tumor ROIs on multiple slices were pooled together and the median was determined. Besides the absolute feature values, the relative feature values were also evaluated, where the parameter value was normalized using the median of contralateral normal-appearing white matter ROI. Interobserver consistency was assessed using intraclass correlation coefficient (ICC) with ICC >0.80, excellent; 0.61–0.80, good; 0.41–0.60, moderate; 0.21–0.40, fair; and <0.20, poor agreement (20). The normality of the distribution of all parameters was analyzed by the Kolmogorov–Smirnov test. Mann–Whitney U test was used to assess the difference of parameter values between recurrent tumor group and treatment response group. The receiver operating characteristic (ROC) curves of all parameters were obtained and the areas under the ROC curves (AUC) were evaluated to determine the discriminating power of DCE parameters between recurrent tumor group and treatment response group. Optimal cut-off values were chosen using the Youden index on the ROC curves, and the corresponding statistical metrics (sensitivity, specificity, accuracy) were computed. To account for the unbalanced issue in the data, the bootstrap re-sampling technique was employed in the study, where re-sampling with replacement was utilized to create a new dataset from original dataset but with predesigned balanced data size, followed by ROC analysis for the resampled dataset, and the process was repeated 200 times, with AUCs (mean ± standard deviation) being calculated. The false discovery rate (FDR) was used to obtain adjusted P values which correct for multiple testing when comparing the various parameters. P<0.05 indicates statistical significance. Statistical analyses were performed using MATLAB (2020b, MathWorks, Natick, MA).

## 3 Results

### 3.1 Baseline characteristics of the participants

A total of 52 patients were included, where 40/52 (76.9%) were recurrent and 12/52 (23.1%) had treatment response. Of all patients, 40 were confirmed by follow-up and 12 were confirmed by surgery. The demographic and clinical characteristics of the patient cohort were shown in **Table 1**.

**Table 1:** The demographic and clinical characteristics of the patients.

Variable	Recurrent (n=40)	Treatment response (n=12)	*P* value
Grouping criteria			0.433
Follow-up	30	10	
Pathology	10	2	
Age	56.7 ± 8.33	52.9 ± 14.58	**0.011**
Sex			0.746
Male	20	5	
Female	20	7	
Cerebral lobe			0.304
Frontal lobe	15	5	
Parietal lobe	5	4	
Occipital lobe	7	0	
Temporal lobe	12	3	
Others	1	0	
Location			1.000
Left	19	6	
Right	21	6	
Lesion number			0.743
Single	19	7	
Multiple	21	5	
WHO grade			**0.011**
Grade 3	3	5	
Grade 4	37	7	
Integrated classification			0.121
Glioblastoma, IDH wild-type	33	7	
Astrocytoma, IDH wild-type	2	0	
Astrocytoma, IDH mutant	2	2	
Astrocytoma, NOS	1	2	
Oligodendroglioma, IDH wild-type	1	0	
Oligodendroglioma, IDH mutant	1	0	
Oligodendroglioma, NOS	0	1	
IDH mutation status			**0.018**
Mutant	3	2	
Wild-type	36	7	
NA	1	3	

Bold *P* values less than 0.05 indicate a statistically significant difference. IDH, isocitrate dehydrogenase; NOS, not otherwise specified.

### 3.2 Intraclass correlation coefficients

**Supplementary Table SA2** showed the ICC values for the measured parameters of DP with delineation in anatomical images and parameter heat maps, where most ICC values in both delineation methods were greater than 0.9, indicating excellent agreement between measurements from two observers. Hence, the parameter values as measured by two observers were averaged and utilized in the subsequent analysis.

### 3.3 Comparison of kinetic parameter values between MRI scanners

To compare the difference between MRI scanners, **Supplementary Table SA3** presented the measured values of kinetic parameters (median followed by interquartile range) by different TKMs, where *P* values of DP-derived parameters were mostly greater than 0.05 except for V_e_, indicating only the difference of V_e_ was statistically significant between two scanners in DP model. Nevertheless, *P* values of other kinetic parameters by other TKMs were largely less than 0.05, indicating significant difference between scanners.

The normalized kinetic parameter values were listed in **Table 2**, where *P* values of all kinetic parameters by all TKMs were greater than 0.05, suggesting that the difference on the normalized kinetic parameter value between two scanners was insignificant. Hence, the following analysis was largely based on the normalized kinetic parameter values.

**Table 2:** Comparison between MRI scanners on normalized kinetic parameter values in recurrent glioma tissue.

Parameters	United Imaging (n = 30)	Philips (n = 10)	*P* value
DP			
CBF	1.03 (0.88: 1.20)	0.99 (0.84: 1.22)	0.79
MTT	4.85 (2.56: 6.12)	5.43 (2.52: 9.48)	0.57
V_p_	4.14 (2.40: 6.54)	4.44 (2.82: 6.95)	0.59
V_e_	41.89 (26.53: 69.46)	74.30 (55.37: 106542.51)	0.11
PS	42.35 (17.83: 112.47)	38.46 (20.67: 27965.72)	0.24
E	43.64 (19.51: 94.95)	40.04 (16.37: 27777.53)	0.14
TH			
CBF	1.52 (1.06: 2.54)	1.34 (1.03: 1.87)	0.83
MTT	4.11 (1.65: 5.98)	6.60 (3.50: 9.34)	0.11
V_p_	4.72 (3.10: 7.91)	7.20 (5.87: 8.57)	0.37
V_e_	40.16 (26.10: 79.80)	64.23 (43.14: 13248755.01)	0.09
PS	25.64 (10.53: 56.83)	54.54 (16.26: 926318.71)	0.17
E	14.59 (7.37: 23.85)	25.02 (10.39: 1447508.26)	0.11
Brix			
CBF	1.54 (1.14: 1.89)	1.28 (1.06: 1.98)	0.25
MTT	11.11 (7.76: 17.98)	17.94 (11.83: 26.70)	0.50
V_p_	18.60 (9.03: 30.59)	22.94 (12.61: 39.74)	0.74
V_e_	49.09 (27.33: 85.92)	46.61 (24.20: 86.11)	0.11
PS	32.60 (12.90: 54.18)	37.33 (23.29: 72.01)	0.09
E	20.22 (11.38: 39.10)	27.97 (16.82: 46.87)	0.11
ETM			
K^trans^	1.95 (0.79: 5.71)	3.96 (1.04: 13.15)	0.55
V_e_	245.42 (46.55: 7925.95)	133.37 (53.31: 18122.45)	0.94
K_ep_	0.14 (0.01: 0.27)	0.17 (0.01: 0.26)	0.37
V_p_	3.17 (1.60: 6.00)	4.09 (2.10: 5.86)	0.33

CBF, cerebral blood flow; MTT, mean transit time; V_p_, plasma volume; V_e_, extravascular volume; PS, vascular permeability; E, first-pass extraction ratio.

### 3.4 Differential diagnosis between recurrent tumor and treatment response

**Figure 3:**
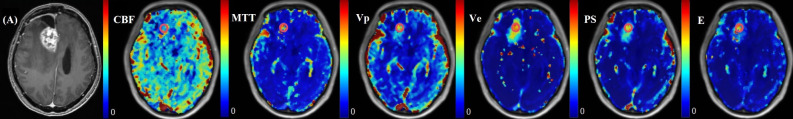
A 36-year-old female with WHO grade 4 glioblastoma of the right frontal lobe. A new enhanced lesion emerged during the follow-up. Postoperative pathology confirmed the enhanced lesion as recurrence. Parametric maps of cerebral blood flow CBF, mean transit time MTT, plasma volume V_p_, extravascular volume V_e_, vascular permeability PS, and first-pass extraction ratio E as derived using DP model.

**Figure 4:**
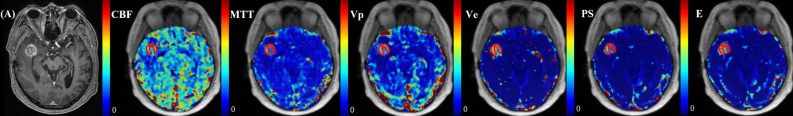
A 57-year-old male with WHO grade 3 oligodendroglioma of the right temporal lobe. Postoperative pathology confirmed the enhanced lesion as radiation necrosis (RN). Parametric maps of blood flow CBF, mean transit time MTT, fractional volume of intravascular space V_p_, fractional volume of interstitial space V_e_, vessel permeability PS, and extraction ratio E as derived using DP model.

The normalized kinetic parameter values of recurrent tumor and treatment response by four TKMs were shown in **Table 3**. Compared with treatment response, lesions with tumor recurrence had higher MTT and V_p_ using advanced TKMs (DP, TH, Brix) (*P*<0.05). As for ETM, V_e_ (*P* = 0.03) and V_p_ (*P* = 0.02) were lower for patients with treatment response compared with patients with tumor recurrence. **Figures 3**, **4** illustrated the parameter maps of a cases of postoperative recurrence and a case of treatment response of glioblastoma based on DP model respectively, where tumor recurrence was manifested on the parameter maps as higher perfusion and higher permeability compared to treatment response.

**Table 3:** Summary of normalized kinetic parameter values (median and interquartile range) of recurrent tumor and treatment response by four TKMs.

Parameters	Recurrent tumor (n=40)	Treatment response (n=12)	*P* value
DP			
CBF	1.00 (0.87: 1.21)	0.95 (0.81: 1.15)	0.38
MTT	4.85 (2.54: 6.79)	1.55 (1.31: 2.37)	**<0.01**
V_p_	4.14 (2.41: 6.63)	1.51 (1.27: 3.16)	**<0.01**
V_e_	47.01 (29.99: 77.22)	33.85 (9.81: 8400.57)	0.23
PS	42.35 (19.25: 133.96)	37.95 (15.72: 314.18)	0.72
E	42.04 (17.94: 114.67)	46.04 (17.95: 2655.28)	0.99
TH			
CBF	1.42 (1.05: 2.39)	1.74 (1.10: 1.95)	0.82
MTT	4.40 (2.19: 7.38)	1.49 (1.07: 2.22)	**<0.01**
V_p_	5.83 (3.53: 8.49)	3.19 (1.68: 4.47)	**<0.01**
V_e_	46.10 (29.25: 88.10)	35.63 (12.49: 144.96)	0.31
PS	28.62 (11.04: 66.55)	29.33 (3.44: 79.16)	0.45
E	14.59 (8.11: 30.77)	10.27 (2.93: 46.27)	0.39
Brix			
CBF	1.48 (1.13: 1.92)	1.17 (0.83: 1.85)	0.24
MTT	11.98 (8.33: 19.48)	4.53 (3.35: 14.39)	**0.02**
V_p_	18.92 (9.57: 31.25)	7.92 (4.11: 10.67)	**<0.01**
V_e_	49.09 (26.09: 86.02)	18.63 (12.10: 40429.65)	0.10
PS	35.19 (15.16: 61.08)	19.04 (3.47: 294.36)	0.37
E	23.54 (12.73: 42.52)	35.92 (4.39: 415.56)	0.94
ETM			
K^trans^	2.87 (0.89: 7.69)	2.08 (0.25: 4.88)	0.38
V_e_	144.59 (45.94: 12718.69)	38.90 (5.69: 129.81)	**0.03**
V_p_	3.17 (1.82: 6.18)	1.33 (0.75: 3.48)	**0.02**
K_ep_	0.17 (0.01: 0.28)	0.17 (0.04: 1.05)	0.43

Bold *P* values less than 0.05 indicate a statistically significant difference. CBF, cerebral blood flow; MTT, mean transit time; V_p_, plasma volume; V_e_, extravascular volume; PS, vascular permeability; E, first-pass extraction ratio.

**Figure 5:**
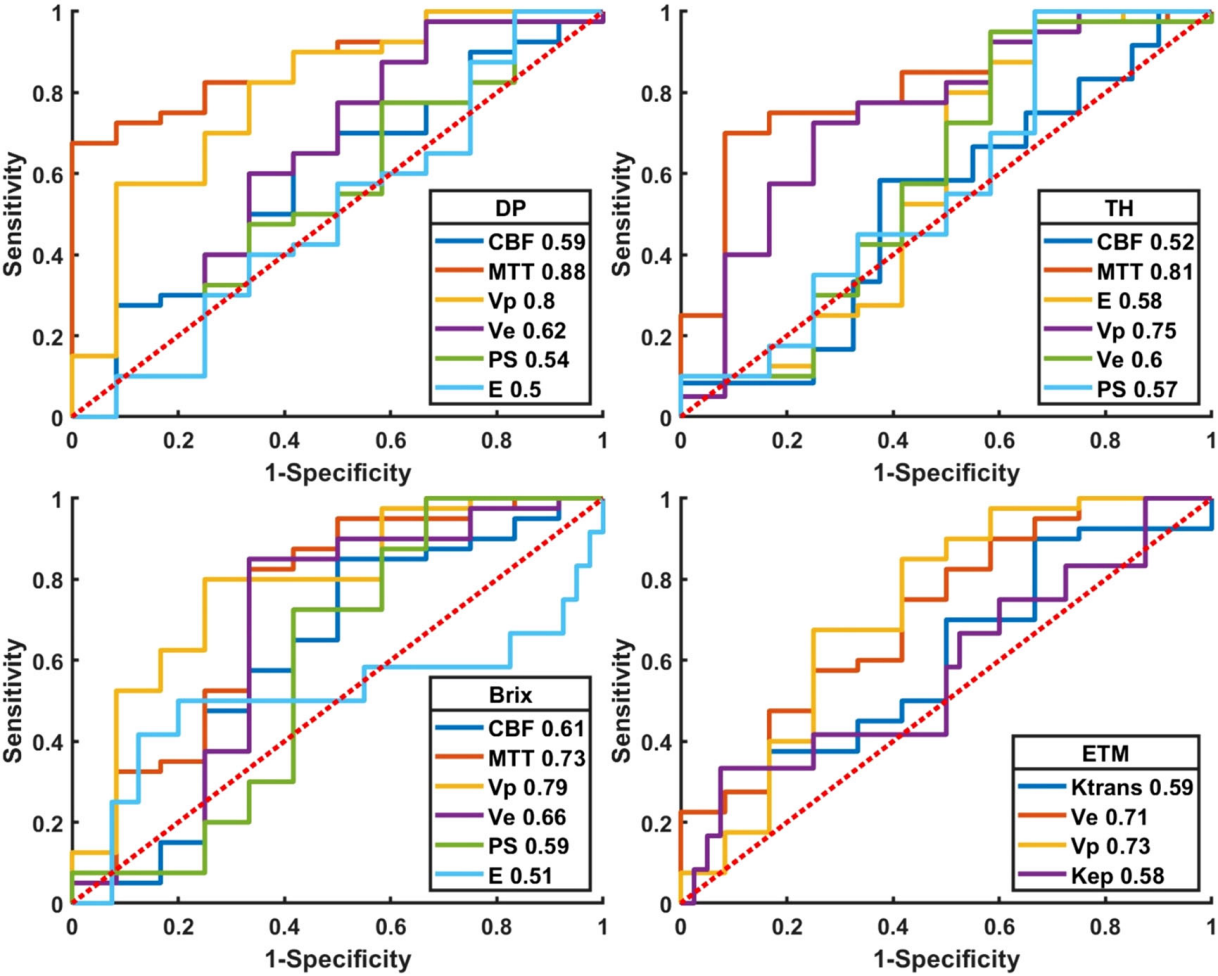
Plot of ROCs of normalized kinetic parameters derived by four tracer kinetic models in differentiating HGGs recurrence from treatment response. DP: distributed parameter model, TH: tissue homogeneity model, Brix: Brix’s conventional two-compartment model, ETM: extended-Tofts model. Regions of interest (ROI) were delineated on kinetic parameter heat maps.

Quantitative diagnostic metrics of normalized kinetic parameters derived by four TKMs were shown in **Figure 5** (plot of the ROC curves) and **Table 4** (optimal cutoff, AUC values, sensitivity, specificity, accuracy), where ROIs were delineated based on parameter heat maps. MTT(DP) attained the best performance in all TKMs’ parameters with AUC 0.88, optimal threshold 2.64, specificity 0.92, accuracy 0.77 and sensitivity 0.73. MTT(TH), V_p_ (DP) and V_p_ (Brix) had AUCs around 0.80 (0.81, 0.80, 0.79 respectively), with optimal thresholds of 2.25, 2.02, and 8.90 respectively. The best performance in ETM was V_p_ with AUC 0.73.

**Table 4:** ROC quantitative parameters of normalized kinetic parameters derived by four TKMs in differential diagnosis between recurrent HGG and treatment response, where ROIs were delineated on kinetic parameter heat maps.

Parameters	AUC	Threshold	Sensitivity	Specificity	Accuracy
DP					
CBF	0.59	0.97	0.65	0.58	0.63
MTT	**0.88**	2.64	0.73	0.92	0.77
V_p_	0.80	2.02	0.83	0.67	0.79
V_e_	0.62	40.7	0.60	0.67	0.62
PS	0.54	50.49	0.48	0.67	0.52
E	0.50	38.49	0.58	0.50	0.56
TH					
CBF	0.52	1.71	0.58	0.63	0.62
MTT	0.81	2.25	0.75	0.83	0.77
V_p_	0.75	3.92	0.73	0.75	0.73
V_e_	0.60	31.91	0.73	0.50	0.67
PS	0.57	38.33	0.45	0.67	0.50
E	0.58	7.27	0.80	0.50	0.73
Brix					
CBF	0.61	1.05	0.85	0.50	0.77
MTT	0.73	7.72	0.83	0.67	0.79
V_p_	0.79	8.90	0.80	0.75	0.79
V_e_	0.66	20.57	0.85	0.67	0.81
PS	0.59	20.78	0.73	0.58	0.69
E	0.51	50.00	0.50	0.80	0.73
ETM					
K^trans^	0.59	1.20	0.70	0.50	0.65
V_e_	0.71	46.17	0.75	0.58	0.71
K_ep_	0.58	0.14	0.67	0.48	0.52
V_p_	0.73	2.44	0.68	0.75	0.69

Bold MTT (DP) attained the best performance with the best AUC = 0.88.

CBF, cerebral blood flow; MTT, mean transit time; V_p_, plasma volume; V_e_, extravascular volume; PS, vascular permeability; E, first-pass extraction ratio.

**Figure 6:**
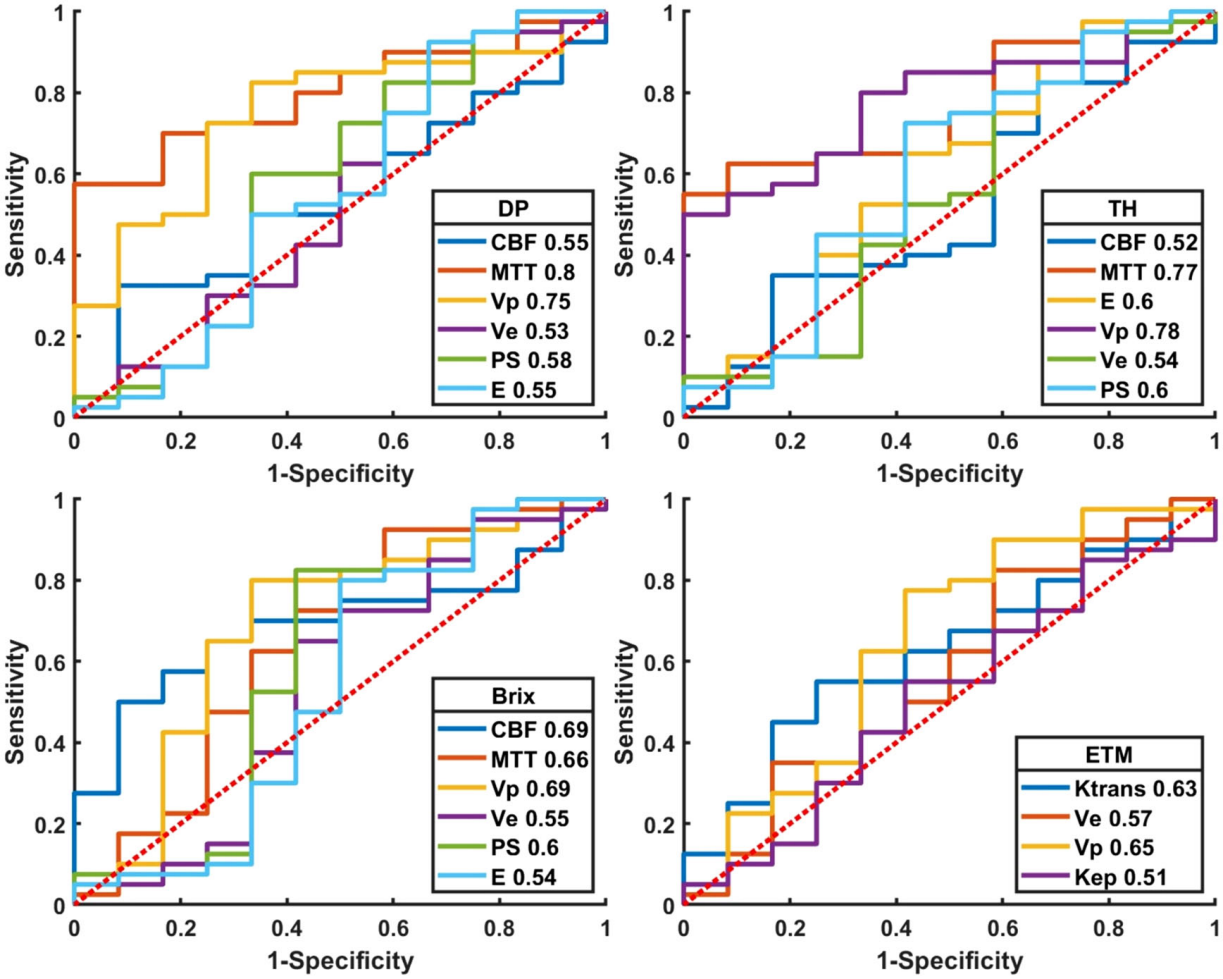
Plot of ROCs of normalized kinetic parameters derived by four tracer kinetic models in differential diagnosis between recurrent HGG and treatment response. DP: distributed parameter model, TH: tissue homogeneity model, Brix: Brix’s conventional two-compartment model, ETM: extended-Tofts model. Regions of interest (ROI) were delineated on structure images.

**Figure 6** and **Supplementary Table SA4** presented the ROC curves and corresponding metrics of normalized kinetic parameters derived by four TKMs, where ROIs were delineated based on structural images. MTT(DP) exhibited the largest AUC (0.80) in all TKMs’ parameters, with optimal cutoff 2.27, specificity 0.83, accuracy 0.73 and sensitivity 0.70. AUCs of ETM parameters were less than 0.66.

The results of bootstrapping test were summarized in **Supplementary Table SA5**, where table entries represented AUCs (mean ± standard deviation) of normalized kinetic parameters. The DP-derived MTT showed the largest AUC (0.88 ± 0.05). TH derived MTT, V_p_ in DP and Brix attained AUCs around 0.80 separately. AUCs by ETM derived parameters were less than 0.75.

## 4 Discussion

This paper presented application of advanced TKMs to the differential diagnosis of tumor recurrence and treatment response in HGGs. MTT and V_p_ of advanced TKMs were higher in recurrent tumor than in treatment response. MTT(DP) attained the largest AUC (0.88). No statistical significance was observed on permeability parameters. Comparatively, advanced TKMs demonstrated advantages over ETM in differentiating glioma recurrence and treatment response.

Previous studies (21–23) on differential diagnosis between recurrent glioma and treatment response using DCE-MRI were largely based on conventional TKMs, which characterize the transport of contrast agent using K^trans^ and are recommended in the Quantitative Imaging Biomarkers Alliance (QIBA) Profile (24), where it is claimed that measured change in K^trans^ of a brain lesion of 21% or larger from DCE-MRI data at 1.5T indicates that a true change has occurred with two-sigma confidence (95%) confidence. In theory, K^trans^ is defined as the exchange rate of contrast agent from the blood vessels into the surrounding interstitial space and represents an important parameter in conventional TKMs. Zahra et al. reviewed 29 studies (total of 1194 patients) that correlate DCE-MRI with histopathological or clinical outcome data relevant to radiotherapy, and found the apparent discrepancy among the reported outcomes, which could be attributed to the heterogeneity in the methods, including the selection of the ROIs and the acquisition and analysis of the DCE-MRI data, as well as the small numbers of patients recruited in some studies (25). O'Connor and coauthors reviewed the role of DCE-MRI for decision making during the drug-development process in about 100 early-phase clinical trials and investigator-led studies of targeted antivascular therapies and found that, unlike serological assays, K^trans^ often had variable meanings between different clinical studies and within one study at different time points, which hindered wider application and acceptance of DCE-MRI in clinical practice (26). The precise meaning of K^trans^ has been theoretically investigated in (27, 28). It is understood that the physiological significance is tissue dependent; if the contrast uptake is flow limited, then K^trans^ will indicate the tissue perfusion, whereas if the uptake is permeability limited, then K^trans^ indicates the permeability. In most cases, K^trans^ indicates a combination of the blood flow and vessel wall permeability properties of tissue. The AUC of normalized K^trans^ (ETM) was 0.59 in differentiating recurrent tumor from treatment response in our study, which corroborated the previous studies (AUCs 0.62 and 0.51, respectively) (29, 30).

The primary mechanisms underlying treatment-related responses in HGGs, including RN and PsP, involve radiation-induced direct damage, injury to vascular endothelial cells, and excessive vascular proliferation or rupture leading to hemorrhage and plasma protein extravasation, which disrupt the blood-brain barrier (BBB), and cell death releases cytokines (e.g., IL-6, TNF-α) and mediators, triggering an inflammatory response that activates cells (31), all of which may contribute to increased vascular permeability. Due to the infiltrative growth characteristics of gliomas, residual tumor cell proliferation can lead to tumor recurrence. Tumor cells secrete pro-angiogenic factors, inducing abnormal vascular proliferation with incomplete basement membranes, resulting in contrast agent extravasation. In summary, the enhancing lesions in treatment-related responses (PsP and RN) primarily arise from therapy-induced inflammatory reactions and vascular permeability changes, whereas tumor recurrence-driven enhancement is driven by tumor cell proliferation and abnormal angiogenesis (32, 33). Consequently, both tumor recurrence and treatment-related responses in HGGs can exhibit elevated permeability parameter values on DCE MRI, which explains the lack of significant statistical differences in permeability parameters between the two groups in this study. Larsen et al.'s study also indicated that BBB permeability parameters could not effectively distinguish between PsP and recurrence (34), which was consistent with our study. Besides, Manual delineation of enhancing regions may include areas of coexisting treatment response and tumor recurrence, particularly in infiltrative gliomas, obscuring true permeability differences. The non-significant permeability results highlight the complexity of PsP/recurrence pathophysiology and the limitations of parameter imaging. This underscores the necessity of combining permeability data with systemic inflammatory markers (e.g., NLR, SII) and volumetric analyses to enhance diagnostic precision (6).

Quantitative interpretation of kinetic parameter maps has two approaches in practice, namely, delineation based on anatomical images or parameter heat maps. The former approach defines lesions and their boundaries from correlative routine scans which have higher spatial resolution in interpreting tissue structures and are acquired in the same imaging plane as DCE-MRI (with similar FOV and spatial coverage) such as T_2_WI and T_1_CE images. This approach has been recommended by the committee of QIBA (24) for reproducibility. In the recent guidelines of both National Comprehensive Cancer Network (NCCN) and European Association of Neuro-Oncology (EANO), perfusion maps (in particular, the map of cerebral blood volume) are recommended to define metabolic hotspots for specific tumor tissue sampling (35, 36). This study compared these two approaches to lesion ROI delineation and demonstrated that parameter heat maps could be more accurate in distinguishing recurrent tumor from treatment response in high-grade glioma patients. The reason might be the heterogeneity of suspicious lesion as delineated in anatomical images, which could compromise the subsequent differential diagnosis where the information was based on the measured parameter values of the registered ROI, likely a mixture of heterogeneous tissue. Comparatively, delineation based on parameter heat maps could yield an ROI with more homogeneous tissue.

As discussed in the latest review paper (16), both MR imaging hardware and the theory of DCE tracer kinetic modeling have undergone significant advances over the years, thereby allowing acquisition of DCE images with higher temporal resolution, better signal-to-noise ratio, wider brain coverage and increased spatial resolution, and enabling separate quantification of CBF and PS in advanced TKMs. This study demonstrated clearly the advantages of advanced TKMs over ETM in differential diagnosis of recurrent tumor and treatment response.

A long-standing challenge in DCE-MRI is the reproducibility of quantitative results across imaging platforms. Standardization of imaging protocol and data post-processing is essential to achieve the purpose. Towards that end, QIBA has recommended the following protocol: (1) using 3D fast spoiled gradient recalled echo sequence; (2) using variable flip angle for T1-mapping measurement; (3) scanning parameters stay constant; (4) dynamic scan duration up to 6 mins; (5) temporal resolution less than 5 seconds. This study acquired data using MR scanners from two vendors, but the imaging protocol has been attempted to follow the same standard, as largely recommended by QIBA, with the equivalent sequence, the same temporal resolution and the same brain coverage. Though some measured values in some TKMs showed significant difference between scanners, it turned out that the difference of the normalized value is statistically insignificant, which indicated the potential for the imaging protocol and the current DCE-MRI processing flow to fulfill the promise of using DCE-MRI as a clinically useful tool.

There are several limitations in our study. First, this was a single-center retrospective study with a moderate sample size. Second, the delineation of ROI was subjective, and the results might be biased, especially for lesions with unclear enhancement. Third, portion of data was evaluated based on follow-up results of imaging and clinical signs, which might be different from histopathological results.

## 5 Conclusion

In differentiating recurrence and post-treatment response in HGGs, DP demonstrated the best performance, with parameter MTT having the highest diagnostic performance. Moreover, MTT(TH) and V_p_ (DP, Brix) could also serve as potential quantitative imaging biomarkers. The kinetic parameters derived by advanced TKMs yielded superior performance compared to those by conventional ETM. Interpretation of TKM parameters in terms of treatment response assessment was best performed in the heat maps of kinetic parameters.

## Data Availability

The original contributions presented in the study are included in the article/**Supplementary Material**. Further inquiries can be directed to the corresponding author.
