# Supplementary material for: The diagnostic value of advanced tracer kinetic models in evaluating high grade gliomas recurrence and treatment response using dynamic contrast-enhanced MRI
Source: Front Oncol. 2025 Apr 16;15:1536122. doi: 10.3389/fonc.2025.1536122 (PMC12043873; doi:10.3389/fonc.2025.1536122)
Supplement: Supplementary file 1 [file DataSheet1.docx]

**SUPPLEMENTAL FILES**

**Table A1.** The parameters of MRI.

| Scanner | Sequence | Slice  Thickness  (mm) | Voxel  (mm*mm*mm) | FOV  (mm*mm) | TR  (ms) | TE  (ms) | Flip angle  (°) |
| --- | --- | --- | --- | --- | --- | --- | --- |
| uMR790  (3.0 T) | T_1_WI | 1 | 1 * 1 * 1 | 256 * 232 *176 | 7.9 | 3.1 | 10 |
|  | T_2_WI | 1 | 1 * 1 * 1 | 256 * 232 *176 | 2200 | 606.4 | 90 |
|  | T_2_-FLAIR | 1.08 | 1.07*1.07*1.08 | 256 * 232 *176 | 4800 | 428 | 40 |
|  | T_1_CE | 1 | 1 * 1 * 1 | 256 * 232 *176 | 7.9 | 3.1 | 10 |
|  | DCE | 5 | 1.5 * 1.5 * 5 | 240 * 220 *100 | 3.47 | 1.9 | 13 |
| Philips Ingenia CX  (3.0 T) | T_1_WI | 1 | 1 * 1 * 1 | 240 * 240 *180 | 6.6 | 3.0 | 8 |
|  | T_2_WI | 1 | 1 * 1 * 1 | 250 * 250 *180 | 2800 | 280 | 90 |
|  | T_2_-FLAIR | 1.03 | 1.09*1.10*1.10 | 240 *240 *180 | 4800 | 255 | 40 |
|  | T_1_CE | 1 | 1 * 1 * 1 | 240 * 240 *180 | 6.6 | 3.0 | 8 |
|  | DCE | 5 | 1 * 1 * 5 | 220 * 189 *100 | 3.8 | 1.84 | 15 |

**Table A2.** Intraclass correlation coefficients of distributed parameter model parameters between two observers to delineate lesion region of interest in parametric heat map or anatomical image independently.

| Delineation methods | CBF | MTT | Vp | Ve | PS | E |
| --- | --- | --- | --- | --- | --- | --- |
| Parameter heat map | 0.91 | 0.95 | 0.92 | 0.95 | 0.95 | 0.92 |
| Anatomical image | 0.94 | 0.93 | 0.88 | 0.91 | 0.87 | 0.95 |

**Table A3.** Comparison between MRI scanners on measured kinetic parameter values in recurrent glioma tissue

| Parameters | United Imaging (n = 30) | Philips (n = 10) | *P* value |
| --- | --- | --- | --- |
| **DP** |  |  |  |
| CBF | 19.75 (17.54 : 24.31) | 21.49 (20.24 : 24.10) | 0.25 |
| MTT | 9.36 (4.13 : 10.58) | 13.55 (3.56 : 17.22) | 0.33 |
| V_p_ | 2.32 (1.49 : 3.53) | 5.10 (1.71 : 5.93) | 0.09 |
| V_e_ | 17.68 (13.16 : 23.26) | 30.08 (27.49 : 45.82) | <0.01 |
| PS | 4.70 (2.71 : 6.77) | 8.47 (4.92 : 10.88) | 0.07 |
| E | 22.09 (12.37 : 30.63) | 33.58 (18.43 : 39.70) | 0.15 |
| **TH** |  |  |  |
| CBF | 24.04 (17.92 : 26.37) | 29.15 (25.87 : 31.59) | 0.02 |
| MTT | 7.20 (3.64 : 14.32) | 13.43 (7.71 : 18.42) | 0.17 |
| V_p_ | 3.09 (1.68 : 4.88) | 5.81 (4.22 : 7.41) | 0.02 |
| V_e_ | 18.03 (12.53 : 26.39) | 32.62 (29.93 : 40.28) | <0.01 |
| PS | 4.87 (2.98 : 7.16) | 9.16 (6.96 : 10.78) | <0.01 |
| E | 17.27 (12.15 : 22.60) | 29.75 (18.68 : 33.69) | 0.04 |
| **Brix** |  |  |  |
| CBF | 23.34 (17.36 : 31.05) | 32.80 (23.25 : 41.08) | 0.10 |
| MTT | 11.49 (9.54 : 12.91) | 14.60 (9.96 : 20.80) | 0.07 |
| V_p_ | 4.42 (3.00 : 5.98) | 9.06 (5.61 : 11.32) | <0.01 |
| V_e_ | 20.41 (15.28 : 30.07) | 31.73 (27.33 : 36.39) | <0.01 |
| PS | 4.83 (3.01 : 6.79) | 8.05 (5.88 : 9.78) | 0.02 |
| E | 21.28 (14.66 : 26.08) | 21.64 (19.34 : 29.84) | 0.36 |
| **ETM** |  |  |  |
| K^trans^ | 0.04 (0.03 : 0.06) | 0.07 (0.05 : 0.09) | 0.02 |
| V_e_ | 16.55 (10.18 : 23.78) | 28.82 (26.17 : 37.84) | <0.01 |
| K_ep_ | 0.25 (0.18 : 0.31) | 0.23 (0.20 : 0.27) | 0.48 |
| V_p_ | 1.69 (1.14 : 2.30) | 2.05 (1.43 : 2.25) | 0.92 |

**Table A4.** ROC quantitative metrics of normalized kinetic parameters derived by four TKMs in differential diagnosis between recurrent HGG and treatment response, where ROIs were delineated on structure images

| Parameters | AUC | Threshold | Sensitivity | Specificity | Accuracy |
| --- | --- | --- | --- | --- | --- |
| **DP** |  |  |  |  |  |
| CBF | 0.55 | 1.05 | 0.50 | 0.67 | 0.54 |
| MTT | **0.80** | 2.27 | 0.70 | 0.83 | 0.73 |
| V_p_ | 0.75 | 2.57 | 0.73 | 0.75 | 0.73 |
| V_e_ | 0.53 | 37.48 | 0.63 | 0.50 | 0.60 |
| PS | 0.58 | 29.52 | 0.60 | 0.67 | 0.62 |
| E | 0.55 | 30.28 | 0.50 | 0.67 | 0.54 |
| **TH** |  |  |  |  |  |
| CBF | 0.52 | 1.48 | 0.70 | 0.42 | 0.63 |
| MTT | 0.77 | 1.61 | 0.63 | 0.92 | 0.69 |
| V_p_ | 0.78 | 2.25 | 0.80 | 0.67 | 0.77 |
| V_e_ | 0.54 | 24.82 | 0.80 | 0.42 | 0.71 |
| PS | 0.60 | 14.35 | 0.73 | 0.58 | 0.69 |
| E | 0.60 | 6.36 | 0.65 | 0.58 | 0.63 |
| **Brix** |  |  |  |  |  |
| CBF | 0.69 | 0.94 | 0.65 | 0.75 | 0.67 |
| MTT | 0.66 | 5.50 | 0.73 | 0.58 | 0.69 |
| V_p_ | 0.69 | 4.90 | 0.80 | 0.67 | 0.77 |
| V_e_ | 0.55 | 41.54 | 0.65 | 0.58 | 0.63 |
| PS | 0.60 | 10.16 | 0.83 | 0.58 | 0.77 |
| E | 0.54 | 12.01 | 0.80 | 0.50 | 0.73 |
| **ETM** |  |  |  |  |  |
| K^trans^ | 0.63 | 3.75 | 0.55 | 0.75 | 0.60 |
| V_e_ | 0.57 | 20.81 | 0.83 | 0.42 | 0.73 |
| K_ep_ | 0.51 | 0.16 | 0.55 | 0.58 | 0.56 |
| V_p_ | 0.65 | 2.08 | 0.78 | 0.58 | 0.73 |

**Table A5.** AUC values (mean ± standard deviation) of normalized kinetic parameters by four TKMs in differential diagnosis between recurrent tumor and treatment response after bootstrap resampling with 200 repetitions.

| Parameter\Model | DP | TH | Brix | ETM |
| --- | --- | --- | --- | --- |
| CBF | 0.60 ± 0.07 | 0.57 ± 0.06 | 0.63 ± 0.08 |  |
| MTT | **0.88 ± 0.05** | 0.81 ± 0.08 | 0.73 ± 0.08 |  |
| V_p_ | 0.80 ± 0.07 | 0.75 ± 0.08 | 0.79 ± 0.08 | 0.72 ± 0.08 |
| V_e_ | 0.63 ± 0.08 | 0.63 ± 0.08 | 0.67 ± 0.09 | 0.73 ± 0.08 |
| PS | 0.58 ± 0.06 | 0.60 ± 0.07 | 0.60 ± 0.07 |  |
| E | 0.57 ± 0.05 | 0.61 ± 0.07 | 0.58 ± 0.05 |  |
| K^trans^ |  |  |  | 0.60 ± 0.07 |
| K_ep_ |  |  |  | 0.62 ± 0.08 |
